# THE GOOD, THE BAD, AND THE UGLY: EXAMINING OUTCOMES OF TECHNOLOGY USE AMONG OLDER ADULTS

**DOI:** 10.1093/geroni/igz038.699

**Published:** 2019-11-08

**Authors:** Shelia Cotten, Shelia R Cotten, Travis Kadylak

**Affiliations:** Michigan State University, East Lansing, Michigan, United States

## Abstract

Older adults are increasingly using information and communication technologies (ICTs) to communicate with social ties, gather information to make decisions, and for entertainment purposes. Research is increasingly showing that using ICTs has a range of potential benefits for older adults. However, less research examines the potential negative outcomes of ICT use for older adults. Data from a nationally representative sample of older adults in the United States is used to examine positive and negative outcomes of ICT use. Traditional well-being and social connection outcomes are examined as well as new stressors associated with mobile phone use. Our findings suggest that ICT use has varying effects on older adults, depending upon the type, level, and purposes of use. Implications are discussed for entities seeking to encourage ICT use to enhance health and quality of life among older adults.

